# Is Triglyceride-Glucose Index a Valuable Parameter in Peripheral Artery Disease?

**DOI:** 10.7759/cureus.35532

**Published:** 2023-02-27

**Authors:** Serhat Caliskan, Ferit Boyuk

**Affiliations:** 1 Cardiology, Bahçelievler State Hospital, Istanbul, TUR; 2 Cardiology, Health Sciences University, Yedikule Chest Diseases and Thoracic Surgery Training and Research Hospital, Istanbul, TUR

**Keywords:** intermittent claudication, doppler ultrasonography, insulin resistance, triglyceride-glucose index, peripheral artery disease

## Abstract

Background

The aim of this study was to investigate the relationship between the triglyceride-glucose (TyG) index and peripheral artery disease.

Methodology

This was a single-center, observational, retrospective study that included patients evaluated with color Doppler ultrasonography. A total of 440 individuals, 211 peripheral artery patients and 229 healthy controls, were included in the study.

Results

The TyG index levels were significantly higher in the peripheral artery disease group than in the control group (9.19 ± 0.57 vs. 8.80 ± 0.59; p < 0.001). The multivariate regression analysis conducted to determine the independent predictors of peripheral artery disease revealed that age (odds ratio (OR) = 1.111, 95% confidence interval (CI) = 1.083-1.139; p < 0.001), male gender (OR = 0.441, 95% CI = 0.249-0.782; p = 0.005), diabetes mellitus (OR = 1.925, 95% CI = 1.018-3.641; p = 0.044), hypertension (OR = 0.036, 95% CI = 0.285- 0.959; p = 0.036), coronary artery disease (OR = 2.540, 95% CI = 1.376-4.690; p = 0.003), white blood cell count (OR = 1.263, 95% CI = 1.029-1.550; p = 0.026), creatinine (OR = 0.975, 95% CI = 0.952-0.999; p = 0.041), and TyG index (OR = 1.111, 95% CI = 1.083-1.139; p < 0.001) were independent predictors of peripheral artery disease. The cut-off value of the TyG index in predicting peripheral artery disease was determined to be 9.06 with a sensitivity of 57.8% and a specificity of 70% (area under the curve = 0.689; 95% CI = 0.640-0.738; p < 0.001).

Conclusions

High TyG index values can be used as an independent predictor of peripheral artery disease.

## Introduction

Peripheral artery disease is a commonly encountered disease in the population, usually with atherosclerosis as its etiology, with high morbidity and mortality [1]. According to previous studies, its prevalence is estimated to range between 3% and 13% [2]. Because it mostly develops in the background of atherosclerosis, its concomitance with other vascular pathologies such as coronary artery disease and cerebrovascular diseases is common. Its incidence has been increasing with the aging population worldwide. It is a high-cost disease for health systems as it causes the loss of workforce and low quality of life, as well as morbidity and mortality [3].

The risk factors of atherosclerosis such as advanced age, hypertension, hyperlipidemia, diabetes, smoking, and chronic kidney failure are important in the development and course of peripheral artery disease [4]. Particularly, in peripheral artery disease accompanied by diabetes, arterial bed involvement is more commonly seen. In addition, severe pathologies such as extremity amputations are more frequently noted in peripheral artery patients with the diagnosis of diabetes mellitus [5,6]. As a result of insulin resistance, caused by hyperinsulinemia, the release of nitric oxide, a vasodilator, from the endothelium decreases, and the level of endothelin-1, which has vasoconstrictor properties, increases, which accelerates the development of atherosclerosis by contributing to endothelial dysfunction. Moreover, an increase in levels of fibrinogen and thromboxane as well as platelet aggregation is observed in connection with the increased insulin level [7,8].

Many methods are used to measure insulin resistance. Although the gold standard method for the detection of insulin resistance is the hyperinsulinemic-euglycemic clamp technique, it is utilized in clinical studies rather than being used routinely because it is time-consuming and costly to be performed in clinical practice [9]. Due to the difficulties experienced, some inexpensive and practical mathematical formulas have been developed to measure insulin resistance. The most commonly used method is the Homeostasis Model Assessment of Insulin Resistance (HOMA-IR) method which is calculated using fasting serum glucose and fasting serum insulin values. However, the fact that insulin is not studied in primary healthcare centers constitutes the biggest limitation of this method [10]. Thus, a simple and low-cost method that can be used in primary healthcare centers is needed. Hence, the triglyceride-glucose (TyG) index that is measured with the use of triglyceride and fasting blood sugar values has been developed [11]. Studies have demonstrated that insulin resistance measured by the TyG index highly correlates with insulin resistance detected by the HOMA-IR method [12].

Doppler ultrasonography is a rapid, non-invasive test that can be applied bedside in the anatomical evaluation of peripheral arteries and in the determination of the morphology of artery stenosis. It provides anatomical and functional information in peripheral artery disease and has a sensitivity of 92% and a specificity of 97% in the prediction of artery stenosis [13].

The most common symptom seen in peripheral artery disease is intermittent claudication [14]. However, about half of the patients present with asymptomatic or atypical pain [15]. Although it affects the quality of life, causes serious complications, and is associated with mortality, asymptomatic and symptomatic patients are diagnosed late and cannot receive adequate treatment. This situation negatively affects the prognosis of the disease [16]. Previous studies have shown the relationship between the TyG index and atherosclerosis and cardiovascular mortality. In this study, we investigated the role of the TyG index, a practical and inexpensive method, in the early diagnosis and prediction of peripheral artery disease.

## Materials and methods

Study design and participants

This was a single-center, observational, retrospective study of patients with peripheral artery disease. We included 211 consecutive patients with a diagnosis of peripheral artery disease. A total of 229 subjects without peripheral artery disease served as the control group. All included patients visited either the Cardiology or Cardiovascular Surgery department at Bahçelievler State Hospital, Istanbul between October 01, 2017, and October 01, 2022.

At the time of diagnosis, patients under the age of 18, those with malignancy, those with chronic kidney failure and chronic liver disease, pregnant women, those receiving antidiabetic and antihyperlipidemic therapy, and those with active infection or chronic inflammatory disease were not included in the study.

The study conformed to the principles outlined in the Declaration of Helsinki and was approved by the local ethics committee of Istanbul Bakirkoy Dr. Sadi Konuk Training and Research Hospital (05.20.2019, 2019-10-10).

Data collection

Hypertension was defined as systolic blood pressure  ≥140 mmHg or diastolic blood pressure  ≥90 mmHg in clinical measurements or the use of antihypertensive drugs [17]. Diabetes mellitus was defined as a fasting glucose level ≥126 mg/dL (≥7.0 mmol/L) or the use of any antidiabetic drugs [18]. Hyperlipidemia was considered as the patient’s total cholesterol level above 200 mg/dL [19]. History of coronary artery disease was defined by a patient’s self-reported history of myocardial infarction or prior coronary revascularization, and history of stroke was defined as a patient’s self-reported history of stroke. Those who currently smoke and those who quit smoking one month before the study were considered active smokers.

A Toshiba Applio500 (TUS A500) ultrasonography device and an 11 MHz linear probe were used in the arterial Doppler assessment of the lower extremity of the patients. The patients were evaluated from the aorta distal to the ankle after resting for 15 minutes. The proximal assessment of the popliteal artery and trifurcation arteries was performed in the prone position, while other arteries were evaluated in the supine position. Maximum velocities, flow form, and spectral changes were analyzed in the Doppler ultrasonography assessment. The peak systolic velocity ratio was calculated by dividing the maximum velocity at the narrowest part of any stenotic segment by the maximal velocity at 1.5-2 cm proximal to the stenosis. A ratio of 2 or greater was regarded as severe stenosis (over 50%). The diagnosis of occlusion was made in the case of no flow in the artery [20].

Blood samples were taken from the antecubital vein after 12 hours of fasting. The hematological parameters were determined using an XT-4000i Hematology Analyzer (Sysmex, Kobe, Japan). The biochemical analyses to detect alanine aminotransferase, aspartate aminotransferase, creatinine, and lipid profile (total cholesterol, high-density lipoprotein-cholesterol, low-density lipoprotein-cholesterol, and triglyceride levels) were performed with the AU5800 Clinical Chemistry System (Beckman Coulter, Inc., California, USA). The following formula was used to calculate the TyG index: TyG index ln[TG (mg/dL) fasting blood glucose (mg/dL)/2] [21]. Body mass index was calculated using the following formula: weight (kg)/height (m)^2^.

Statistical analysis

All statistical analyses were conducted using IBM SPSS Statistics version 22.0 (IBM Corp., Armonk, NY, USA) and MedCalc version 12.3.0.0 software packages. Normally distributed numerical variables are presented as mean ± standard deviation, whereas those that were not normally distributed are presented as median (minimum-maximum). To compare two independent samples, the independent-sample t-test was used when the normality assumption was met, whereas the Mann-Whitney U test was performed when not. The categorical variables are presented as number and percentage values, and the Pearson chi-square test was employed to compare the groups in terms of categorical variables. Correlations between parameters were determined by the Pearson correlation test for the normally distributed variables and by the Spearman correlation test for the non-normally distributed variables. Univariate and multivariate regression analyses were performed to identify risk factors for peripheral artery disease. Receiver operating characteristic (ROC) analysis was conducted to determine the cut-off positivity value of the TyG index. A p-value of less than 0.05 was considered statistically significant.

## Results

Demographic characteristics and laboratory results of the patients included in our study are presented in Table 1. A total of 440 individuals, 211 patients with peripheral artery disease and 229 control individuals without peripheral artery disease, were included in the study. According to the gender of the patients, 159 (75.4%) patients in the peripheral artery disease group and 114 (49.2%) patients in the control group were male (p < 0.001). The mean age was 69.41 ± 10.61 years for patients with peripheral artery disease and 51.79 ± 15.15 years for the control group (p < 0.001).

**Table 1:** Baseline and laboratory characteristics of the patient and control groups. Median (minimum-maximum), mean ± standard deviation, or n (%) values; p < 0.05. PAD: peripheral artery disease; CAD: coronary artery disease; CVD: cerebrovascular diseases; HDLc: high-density lipoprotein-cholesterol; LDLc: low-density lipoprotein-cholesterol; TC: total cholesterol; TG: triglyceride; CRP: C-reactive protein; WBC: white blood cell; TyG: triglyceride-glucose index

	PAD group (n = 211)	Control group (n = 229)	P-value
Age (years)	69.41 ± 10.61	51.79 ± 15.15	<0.001
Gender, male, n (%)	159 (75.4%)	114 (49.8%)	<0.001
Diabetes mellitus, n (%)	84 (39.8%)	33 (14.4%)	<0.001
Hypertension, n (%)	120 (56.9%)	77 (33.6%)	<0.001
Hyperlipidemia, n (%)	67 (31.8%)	48 (21.0%)	0.01
CAD, n (%)	92 (43.6%)	30 (13.1%)	<0.001
CVD, n (%)	24 (11.4%)	12 (5.2%)	0.019
Glucose	121 (59–245)	96 (71–332)	<0.001
Creatinine (mg/dL)	0.9 (0.42–3.32)	0.75 (0.27–1.70)	<0.001
TC (mg/dL)	207.12 ± 46.54	207.03 ± 39.74	0.893
HDLc (mg/dL)	45.92 ± 35.34	49.47 ± 12.91	0.157
TG (mg/dL)	175 (58–882)	122(30–445)	<0.001
LDLc (mg/dL)	121.62 ± 38.77	124.11 ± 35.02	0.479
WBC (10^3^/mm^3^)	8.16 ± 2.09	7.64 ± 1.84	<0.001
Hemoglobin (g/dL)	13.38 ± 1.99	13.66 ± 1.53	0.107
Platelets (10^3^/mm^3^)	250.34 ± 71.01	250.28 ± 60.31	0.993
CRP (mg/L)	3.20 ± 1.19	2.55 ± 1.42	<0.001
TyG index	9.19 ± 0.57	8.80 ± 0.59	<0.001

The presence of coronary artery disease (p < 0.001), cerebrovascular disease (p = 0.019), diabetes mellitus (p < 0.001), hypertension (p < 0.001), and hyperlipidemia (p = 0.01) was higher in the peripheral artery disease group than in the control group (Table 1).

Serum glucose (121 mg/dL, min-max = 59-245 vs. 96 mg/dL, min-max = 71-332; p < 0.001), creatinine (0.9 mg/dL, min-max = 0.42-3.32 vs. 0.75 mg/dL, min-max = 0.27-1.7; p < 0.001), triglyceride (175 mg/dL, min-max = 58-882 vs. 122 mg/dL, min-max = 30-445; p < 0.001), C-reactive protein (CRP) (3.20 ± 1.19 mg/L vs. 2.55 ± 1.42 mg/L; p < 0.001), white blood cell (WBC) count (8.16 ± 2.09 10^3^/mm^3^ vs. 7.64 ± 1.84 10^3^/mm^3^; p < 0.001), and TyG index (9.19 ± 0.57 vs. 8.80 ± 0.59; p < 0.001) levels were significantly higher in the peripheral artery disease group than in the control group. There were no significant differences between the groups regarding other parameters (Table 1).

The results of the multivariate analysis conducted with the significant parameters found in the univariate analysis that was performed to identify the independent predictors of peripheral artery disease showed that age (odds ratio (OR) = 1.111, 95% confidence interval (CI) = 1.083-1.139; p < 0.001), male gender (OR = 0.441, 95% CI = 0.249-0.782; p = 0.005), diabetes mellitus (OR = 1.925, 95% CI = 1.018-3.641; p = 0.044), hypertension (OR = 0.036, 95% CI = 0.285-0.959; p = 0.036), coronary artery disease (OR = 2.540, 95% CI = 1.376-4.690; p = 0.003), WBC (OR = 1.263, 95% CI = 1.029-1.550; p = 0.026), creatinine (OR = 0.975, 95% CI = 0.952-0.999; p = 0.041), and TyG index (OR = 1.111, 95% CI = 1.083-1.139; p < 0.001) were the independent predictors of peripheral artery disease (Tables 2, 3).

**Table 2:** Univariate logistic regression analysis to identify predictors of peripheral artery disease. CAD: coronary artery disease; CVD: cerebrovascular diseases; WBC: white blood cell; CRP: C-reactive protein; TyG: triglyceride-glucose index

	P-value	Odds ratio	95% CI for EXP(B)	
			Lower	Upper
Age	<0.001	1.100	1.080	1.200
Gender	<0.001	0.324	0.216	0.487
Diabetes mellitus	<0.001	0.928	2.479	6.226
Hypertension	<0.001	2.603	1.769	3.831
Hyperlipidemia	0.010	1.754	1.141	2.698
CAD	<0.001	5.128	3.204	8.210
CVD	0.022	2.321	1.130	4.768
Creatinine	<0.001	60.125	20.799	178.813
WBC	0.006	1.146	1.040	1.264
CRP	<0.001	1.464	1.255	1.707
TyG index	<0.001	3.196	2.225	4.591

**Table 3:** Multivariate logistic regression analysis to identify predictors of peripheral artery disease. CAD: coronary artery disease; CVD: cerebrovascular diseases; WBC: white blood cell; CRP: C-reactive protein; TyG index: triglyceride-glucose index

	P-value	Odds ratio	95% CI for EXP(B)	
			Lower	Upper
Age	<0.001	1.111	1.083	1.139
Gender	0.005	0.441	0.249	0.782
Diabetes mellitus	0.044	1.925	1.018	3.641
Hypertension	0.036	0.522	0.285	0.959
Hyperlipidemia	0.900	0.961	0.513	1.799
CAD	0.003	2.540	1.376	4.690
CVD	0.225	0.562	0.222	1.426
Creatinine	0.041	0.975	0.952	0.999
WBC	0.026	1.263	1.029	1.550
CRP	0.131	1.492	0.888	2.507
TyG index	<0.001	1.111	1.083	1.139

**Figure 1:**
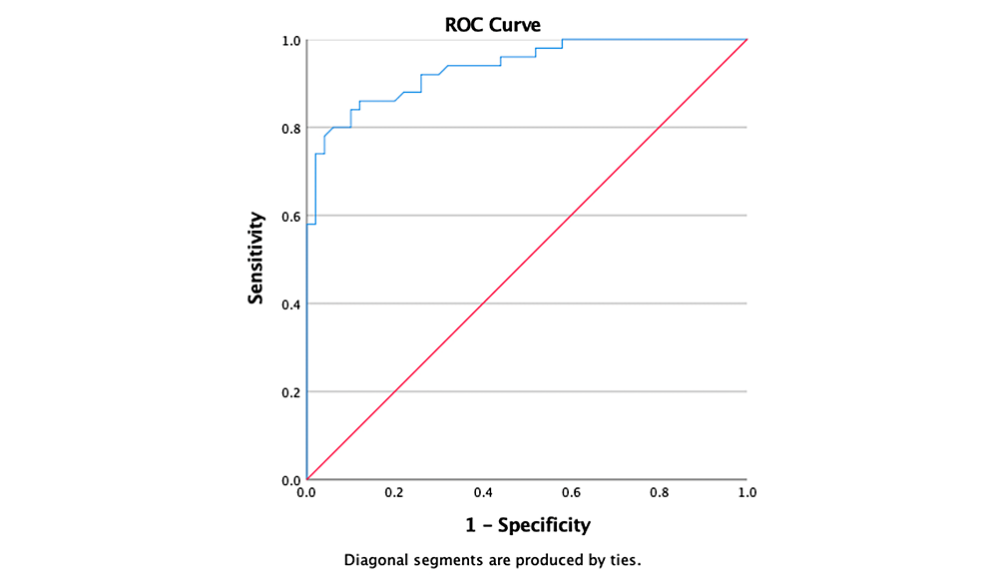
Receiver operator characteristic curve (ROC) of the triglyceride-glucose (TyG) index in predicting peripheral artery disease.

ROC curve analysis was performed to determine the best cut-off value of the TyG index in predicting peripheral artery disease. The cut-off value of the TyG index in the prediction of peripheral artery disease was found to be 9.06 with a sensitivity of 57.8% and a specificity of 70 (area under the curve (AUC) = 0.689; 95% CI = 0.640-0.738; p < 0.001) (Figure 1).

The correlation analysis to evaluate the relationship of the TyG index with clinical and laboratory variables showed that the TyG index was correlated positively with creatinine (r = 0.128, p = 0.007), total cholesterol (r = 0.352, p < 0.001), low-density lipoprotein-cholesterol (r = 0.179, p < 0.001), WBC (r = 0.232, p < 0.001), CRP (r = 0.107, p = 0.024), and age (r = 0.243, p < 0.001), and negatively with high-density lipoprotein (r = -0.411, p < 0.001) (Table 4).

**Table 4:** Correlation between the triglyceride-glucose index and laboratory variables. TC: total cholesterol; HDLc: high-density lipoprotein cholesterol; LDLc: low-density lipoprotein cholesterol; WBC: white blood cell, CRP: C-reactive protein

Variables	r-value	P-value
Creatinine	0.128	0.007
TC	0.352	<0.001
HDLc	-0.411	<0.001
LDLc	0.179	<0.001
WBC	0.232	<0.001
Hemoglobin	0.076	0.110
Platelets	0.073	0.126
CRP	0.107	0.024
Age	0.243	<0.001

## Discussion

The main finding of our study is that the TyG index was higher in patients with peripheral artery disease than in patients in the control group. The relationship between the TyG index and cardiovascular diseases has been previously reported in various studies. In this study, with the use of multivariate analysis, we noted that a high TyG index value was an effective parameter in predicting peripheral artery disease.

Atherosclerosis and the cardiovascular diseases it leads to are the most common causes of death worldwide [22]. Chronic and slowly progressing peripheral artery disease leads to a high cost for health systems with its high prevalence and the consequent loss of workforce [3]. Although different symptoms are observed according to the arterial area involved and the severity of the stenosis, the most frequent symptom is intermittent claudication, which occurs as a consequence of the inability to meet the increased blood demand of the lower extremity muscles during exercise [23]. However, in approximately half of the patients, the disease can manifest asymptomatically or present with atypical complaints [24]. It is crucial to make the diagnosis and begin the treatment at an early stage in patients with atypical complaints or asymptomatic patients to prevent complications and mortality the disease causes. Many parameters have been tested so far for this purpose.

Hyperinsulinemia and increased insulin resistance are known to play a role in the pathophysiology of atherosclerosis through more than one mechanism. These can be listed as the disruption of the balance between nitric oxide and endothelin-1, which are vasodilator and vasoconstrictor substances that play a role in the maintenance of vascular tone in endothelial cells, against nitric oxide; increased chronic inflammation; vascular smooth muscle cell proliferation; and increased connective tissue in the vessel wall [25,26]. Plasma insulin level with its role in the atherosclerotic process constitutes an independent risk factor for cardiovascular diseases [27], which increases the importance of early detection of insulin resistance before cardiovascular diseases are clinically present, and of intervening.

The TyG index, which can be easily calculated from fasting glucose and triglyceride levels, is an effective parameter demonstrating insulin resistance. After being associated with insulin resistance, it has been shown that the TyG index also positively correlates with the development of type 2 diabetes [28]. It is more practical than the HOMA-IR and the hyperinsulinemic-euglycemic clamp test, the gold standard test for determining insulin resistance, because it does not require any additional tests such as insulin level, and because it can be easily calculated using fasting glucose and triglyceride levels, which are routine tests in daily practice. Studies have shown that results of the TyG index and HOMA-IR correlate with the hyperinsulinemic-euglycemic clamp test result [29,30].

The relationship of the TyG index with atherosclerotic cardiovascular diseases has been demonstrated in many studies. In a study investigating the SYNTAX score and major adverse cardiovascular events of 438 patients with non-ST-segment elevation acute coronary syndrome, the TyG index was found to be an independent predictor of cardiovascular outcomes [31]. In another study in which 5,014 patients were followed for 10 years, the TyG index was shown to be related to newly emerging cardiovascular diseases that are independent of other cardiovascular risk factors such as age, gender, smoking, diabetes, and hypertension [32]. A study conducted in South Korea retrospectively evaluating 12,326 asymptomatic adult patients revealed that increased TyG index is an independent predictor of coronary artery calcification progression [33].

In a meta-analysis of eight cohort studies that included 5,731,234 participants with no known prior atherosclerotic cardiovascular disease, high TyG index values were associated with increased stroke, atherosclerotic cardiovascular disease, and coronary artery disease, independent of age, gender, and diabetes status [34]. In this study, we also detected that increased TyG index value is an independent predictor of peripheral artery disease, in line with the literature.

Smoking is one of the most important preventable risk factors that play a role in the development of atherosclerosis. Endothelial damage and inflammation increase as a result of elevated oxidative stress due to smoking [35]. With endothelial damage and the direct effect of oxidative substances, platelet activation and predisposition to thrombosis develop [36]. Smoking also leads to the development of lipid abnormalities and glucose intolerance [37]. Many studies have reported that there is a strong association between smoking and peripheral artery disease depending on the consumed dose, and that continuation of smoking increases peripheral artery disease by two to sixfold [38]. It has been shown that in the follow-up of patients who quit smoking, when compared to patients who continue to smoke, they benefit more from revascularization treatments and there is a significant decrease in mortality rates [39].

Dyslipidemia and hypertension are two important risk factors in the development of atherosclerosis and play an important role in cardiovascular diseases. Clinical studies have revealed that high serum cholesterol levels and hypertension are among the leading risk factors for peripheral artery disease, as in coronary artery disease [40]. Every 40 mg/dL increase in total cholesterol level has been associated with a 1.2-fold increase in the risk of claudication. Similarly, it has been observed that the risk of claudication increases three to fourfold when blood pressure values are above 160/95 mmHg [41]. As hypertension and dyslipidemia frequently coexist, it is recommended to treat the two conditions simultaneously [42].

CRP is an acute-phase protein synthesized in the liver during infection and inflammation, and its role in the course of atherosclerosis, which is an inflammatory process, has been shown in numerous studies [43]. In a study, it was determined that CRP level is a more powerful risk indicator than low-density lipoprotein-cholesterol in terms of cardiovascular events (myocardial infarction, cerebrovascular incidents, coronary revascularization, and death due to cardiovascular causes) [44]. Another study conducted among 144 healthy men followed for 60 months reported that CRP is a predictive indicator for the development of peripheral artery disease [45]. The fact that, in our study, CRP levels were statistically significantly higher in the peripheral artery disease group than in the control group suggests that high CRP levels can be an independent predictor in peripheral artery disease patients, in accordance with other studies in the literature.

Limitations

The biggest limitation of our study is that it was a single-center and retrospective study. Because insulin level was not routinely studied in patients, HOMA-IR values were not calculated, and their comparison with the TyG index results was not performed. Furthermore, grading peripheral artery disease in a larger sample group and revealing its variation with the TyG index could have strengthened our study.

## Conclusions

It is important to screen and detect at-risk individuals before atherosclerosis-related diseases and related complications occur for preventive cardiology. In our study, the TyG index, which is a predictor of atherosclerosis, was found to be significantly higher in patients with peripheral artery disease than those in the control group. These results suggest that patients with a high TyG index value may be at risk for peripheral artery disease. Using the TyG index, patients at risk of peripheral artery disease can be early detected, and a decrease in mortality and morbidity can be achieved by preventing related complications.
